# Is Anopheles gambiae a Natural Host of *Wolbachia*?

**DOI:** 10.1128/mBio.00784-19

**Published:** 2019-06-11

**Authors:** Ewa Chrostek, Michael Gerth

**Affiliations:** a Max Planck Institute for Infection Biology, Berlin, Germany; b Institute of Integrative Biology, University of Liverpool, United Kingdom; University of Texas at Austin

**Keywords:** endosymbionts, malaria, metagenomics, vector biology

## Abstract

*Wolbachia* (*Alphaproteobacteria*, *Rickettsiales*) is an intraovarially transmitted symbiont of insects able to exert striking phenotypes, including reproductive manipulations and pathogen blocking. These phenotypes make *Wolbachia* a promising tool to combat mosquito-borne diseases. Although *Wolbachia* is present in the majority of terrestrial arthropods, including many disease vectors, it was considered absent from Anopheles gambiae mosquitos, the main vectors of malaria in sub-Saharan Africa. In 2014, *Wolbachia* sequences were detected in *A. gambiae* samples collected in Burkina Faso. Subsequently, similar evidence came from collections all over Africa, revealing a high *Wolbachia* 16S rRNA sequence diversity, low abundance, and a lack of congruence between host and symbiont phylogenies. Here, we reanalyze and discuss recent evidence on the presence of *Wolbachia* sequences in *A. gambiae.* We find that although detected at increasing frequencies, the unusual properties of these *Wolbachia* sequences render them insufficient to diagnose natural infections in *A. gambiae*. Future studies should focus on uncovering the origin of *Wolbachia* sequence variants in *Anopheles* and seeking sequence-independent evidence for this new symbiosis. Understanding the ecology of *Anopheles* mosquitos and their interactions with *Wolbachia* will be key in designing successful, integrative approaches to limit malaria spread. Although the prospect of using *Wolbachia* to fight malaria is intriguing, the newly discovered strains do not bring it closer to realization.

## Abstract (precis)

Anopheles gambiae mosquitos are the main vectors of malaria, threatening around half of the world’s population. The bacterial symbiont *Wolbachia* can interfere with disease transmission by other important insect vectors, but until recently, it was thought to be absent from natural *A. gambiae* populations. Here, we critically analyze the genomic, metagenomic, PCR, imaging, and phenotypic data presented in support of the presence of natural *Wolbachia* infections in A. gambiae. We find that they are insufficient to diagnose *Wolbachia* infections and argue for the need of obtaining robust data confirming basic *Wolbachia* characteristics in this system. Determining the *Wolbachia* infection status of *Anopheles* is critical due to its potential to influence *Anopheles* population structure and *Plasmodium* transmission.

## OBSERVATION

*Wolbachia* is an obligate intracellular, intraovarially transmitted bacterium living in symbiosis with many invertebrates (1). Depending on host and symbiont genotypes and environmental conditions, *Wolbachia* has been shown either to affect the biology of its hosts in striking ways or to exert only mild phenotypes. Some of the conspicuous *Wolbachia* phenotypes include reproductive manipulations, where maternally inherited symbionts favor the survival and reproduction of transmitting females over those of noninfected females and nontransmitting males (2). One of the reproductive manipulations, cytoplasmic incompatibility (CI) (3), has been proposed as a tool to suppress mosquito populations and decrease arbovirus burden on humans (4, 5). Bidirectional CI, the inability of females to produce offspring with males harboring a different *Wolbachia* strain, was successful in eliminating the filariasis vector Culex pipiens *fatigans* from Okpo, Myanmar, in 1967 (5) and in suppressing Aedes albopictus, the vector of the dengue, Zika, and West Nile viruses, in recent trials in Lexington, Kentucky, California, and New York, USA (https://mosquitomate.com).

*Wolbachia* can also provide infected individuals with fitness benefits: nutrient provisioning (6), increase in reproductive output (7), and protection against pathogens (8, 9). The last phenotype is also being used to eliminate vector-borne diseases. Aedes aegypti mosquitos artificially transinfected with protective *Wolbachia* organisms are being deployed as a strategy to eradicate dengue virus (10–15). The data from one of the first release sites in Australia suggest that this strategy may limit the number of dengue cases in humans (15).

Malaria is a mosquito-borne disease that threatens around half of the world’s population (16). The potential for the use of *Wolbachia* to block malaria has been recognized since the symbiont’s antiviral and antiparasitic properties were first demonstrated in other insects (8–10, 17). However, *Anopheles* mosquitos were long considered inhospitable for *Wolbachia* (18–20). This started to change in 2006, when *Wolbachia* infections in cultured *Anopheles* cells were established for the first time (21). Next, transient somatic infections were created by intrathoracic inoculation of the virulent *w*MelPop strain of *Wolbachia* into adult mosquitos (22). In somatic transinfections, *Wolbachia* does not infect the germ line (23), which is necessary for its maternal transmission and pathogen blocking-based field applications. Therefore, a successful generation of stable *Wolbachia* infections in Anopheles stephensi by Bian et al. was a big step toward field applications (24). Subsequently, the gut microbiota of A. stephensi and *A. gambiae* were shown to hinder the establishment of heritable *Wolbachia* infections in these species, and curing *Anopheles* of its microbiota enabled *Wolbachia* persistence (25). In 2014, the first evidence for natural *Wolbachia* infections was found in Anopheles gambiae and *Anopheles coluzzii* (two sibling mosquitos species of the Anopheles gambiae species complex, considered the main malaria vectors in sub-Saharan Africa [see Text S1 in the supplemental material for details]) from Burkina Faso (26). This was striking, as the natural *Wolbachia* phenotypes may change mosquito biology and population structure and, as such, affect malaria transmission. Several similar reports identifying *Wolbachia* sequences in *A. gambiae* populations across Africa shortly followed (27–31).

10.1128/mBio.00784-19.2 TEXT S1 Additional text and methods. Download Text S1, PDF file, 0.1 MB. Copyright © 2019 Chrostek and Gerth. 2019 Chrostek and Gerth This content is distributed under the terms of the Creative Commons Attribution 4.0 International license.

Here, we examine the evidence of natural *Wolbachia* infections in Anopheles gambiae mosquitos and screen data from the 1,000 *Anopheles* genomes (Ag1000G) project (32) to reveal that *Wolbachia* reads are extremely rare in this rich and randomized data set. We reanalyze the data from which a genome of the putative *Wolbachia* endosymbiont of *A. gambiae* was assembled (33) to show that the majority of reads in the sample originate from known *Wolbachia* hosts different than *A. gambiae*. Finally, we discuss the requirements to diagnose *Wolbachia* infections in a species previously considered uninfected, the potential ecological interactions which may have led to the observed *Wolbachia* sequence prevalence patterns, and their relevance for the design of successful, integrative approaches to limit malaria spread.

## MOLECULAR EVIDENCE FOR NATURAL *WOLBACHIA* IN ANOPHELES GAMBIAE

The first evidence of natural *Wolbachia* infections in malaria vectors comes from a study on field-collected samples of Anopheles gambiae from Burkina Faso (26), in which *Wolbachia* sequences were detected through 16S V4 amplicon sequencing and a *Wolbachia*-specific PCR targeting the 438-bp *w*Spec region of the 16S rRNA gene sequence (34). Furthermore, whole-genome shotgun sequencing of two ovarian samples was performed. Out of over 164.6 million high-quality *Anopheles*-depleted sequences obtained from two Illumina HiSeq lanes, 571 reads mapped to *Wolbachia* genomes, corresponding to a *Wolbachia* genome coverage of ∼0.05×. Overall, out of an average of over 1,000 *Wolbachia* genes, only 134 had at least one read assigned to them. Moreover, 76 of the 571 reads mapped to *Wolbachia* transposases (26). This demonstrates that the *Wolbachia* sequences in these samples were of extremely low titer; the ratio of *Wolbachia* cell-to-host coverage was ∼1:4,700. For comparison, in various Drosophila melanogaster sequencing projects, observed ratios ranged from 27:1 to 1:5 (35). The data described above represent the only genomic evidence for the presence of *Wolbachia* in *A. gambiae*.

**FIG 1:**
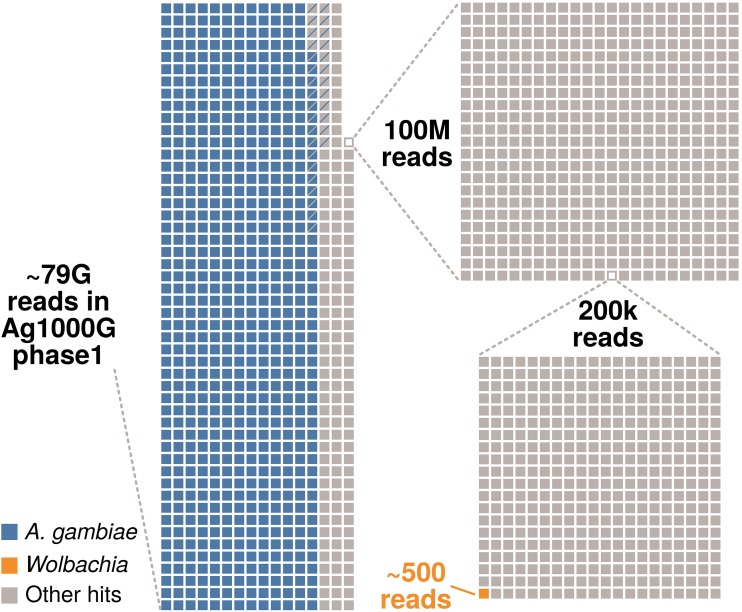
Taxonomic composition of the reads generated in phase 1 of the Ag1000G project. In total, around 79 billion reads were generated from 765 *A. gambiae* mosquitos (32). Around 80% of these reads map to the *A. gambiae* host genome (represented by blue squares on the left). Panels on the right represent sequential magnifications of the portion of non-*Anopheles* reads to visualize the proportion of reads mapping to *Wolbachia*. The proportion of singletons (i.e., reads for which the mate did not map to the same chromosome) for each category are indicated by squares containing diagonal lines. Around 5% of all reads were classified as PCR duplicates but not removed prior to our analysis.

To identify additional *Wolbachia* sequences in *A. gambiae*, we screened data generated in the Ag1000G project, which investigates the genetic variance and population biology of *A. gambiae* (https://www.malariagen.net). We used the data released in the course of phase 1 AR3, namely, Illumina sequences of 765 wild-caught mosquitos from eight African countries (32). Reads for all samples were downloaded from the European Nucleotide Archive (ENA) and mapped to *Wolbachia* reference genomes. Using the criteria of Baldini et al. (26) (see Text S1 for details), we identified 446 reads from 96 libraries as matching *Wolbachia*. In total, there were ∼7.89 × 10^10^ reads across 765 libraries, so only 1 in ∼150 million reads maps to *Wolbachia* (Fig. 1), which corresponds to less than one *Wolbachia* read per sequencing library on average. Furthermore, for all investigated libraries, the reads not mapping to the *A. gambiae* genome were assembled, and the resulting contigs were subjected to a BLAST search against 54 currently available *Wolbachia* assemblies (Text S1). One out of 86,278,186 metagenomic contigs had *Wolbachia* as the best match. Two hundred sixty-four base pairs of this 330-bp contig had ∼91% similarity to the *w*Cle reference genome. As *w*Cle belongs to supergroup F and no other supergroup F sequences have been detected in *A. gambiae* so far, this sequence potentially belongs to *Wolbachia* from a filarial nematode and not to the putative symbiont of *A. gambiae*. Overall, based on a large and broad sampling, our analyses provide independent evidence for only a very sporadic presence, an extremely low titer, or even absence of *Wolbachia* in *A. gambiae*.

**FIG 2:**
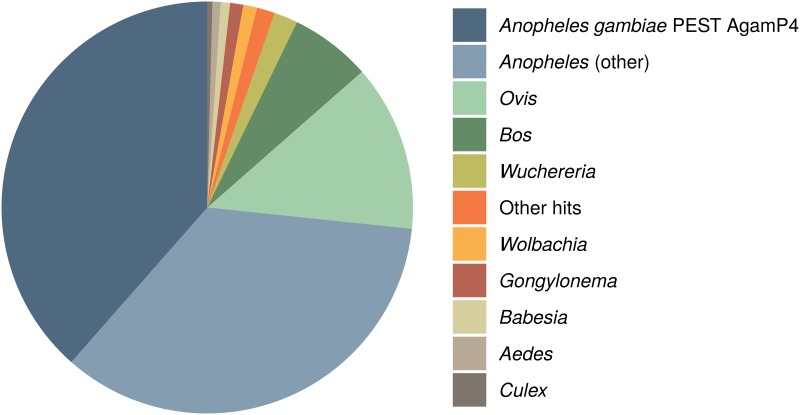
Taxonomic classification of reads in the libraries from which the genome of a putative *Wolbachia* symbiont of *A. gambiae* was assembled (BioSample SAMEA3911293). For more details, refer to Text S1 and Fig. S1 in the supplemental material.

Contrasting with our findings, a recent *in silico* screen of archived arthropod short-read libraries extracted a highly covered *Wolbachia* supergroup B genome from a sample annotated as *A. gambiae* (33). To understand the reasons for this discrepancy, we inspected the sequencing libraries used by Pascar and Chandler (33) and discovered that they contain a mix of sequences of several other potential *Wolbachia* hosts (Fig. 2). Based on the analysis of the ITS2 and COI haplotypes of the most abundant sequences (36, 37), we conclude that the assembled *Wolbachia* genome likely originates from *Anopheles* “species A” and not *A. gambiae* (Fig. 2; Fig. S1; Text S1). Our interpretation is in line with a recent discovery of a highly prevalent supergroup B *Wolbachia* strain, distinct from other supergroup B strains, in *Anopheles* “species A” (31). Our phylogenomic reconstructions further support this, as they place the newly assembled *Wolbachia* genome (33) within supergroup B but separate from most other strains of this lineage (Fig. S1C). These analyses show that unambiguous identification of *Anopheles* species is an additional difficulty in detecting *Wolbachia* infections based on the sequencing data. Therefore, the newly reported genome does not contribute to the understanding of the elusive low-titer *Wolbachia* naturally associated with *A. gambiae*.

10.1128/mBio.00784-19.1 FIG S1 Phylogenetic assessment of SAMEA3911293 taxonomic composition. (A) Phylogenetic reconstruction of *Anopheles* species based on ITS2 alignment of previously published data and all ITS2 contigs present in the meta-assembly of all libraries from SAMEA3911293. Sequences recovered from this library are highlighted in blue. (B) Phylogenetic reconstruction of *Anopheles* based on mitochondrial COI. Sequences from the SAMEA3911293 meta-assembly are highlighted in blue. (C) Phylogeny of *Wolbachia* supergroup B based on concatenated core genome alignments of all strains with a (draft) genome in the NCBI database. Again, the strain isolated from SAMEA3911293 is highlighted in blue. Download FIG S1, PDF file, 0.04 MB. Copyright © 2019 Chrostek and Gerth. 2019 Chrostek and Gerth This content is distributed under the terms of the Creative Commons Attribution 4.0 International license.

The putative low-titer *Wolbachia* infections required improved diagnostics. This prompted Shaw et al. to modify the *w*Spec PCR protocol by including a nested pair of primers and increasing the number of cycles to a total of 72 (nested PCR with 37 cycles in the 1st PCR and 35 cycles in the 2nd PCR, which uses the product of the 1st PCR as a template), potentially amplifying the initial 16S rRNA template over 10^21^ times (28). The protocol was used in several subsequent studies (29–31) but proved unreliable, as Gomes et al. reported 19% of the technical replicates yielding discordant results, even when the total number of cycles was increased to 80 (29). At the same time, the *w*Spec amplification protocol was sensitive enough to detect *Wolbachia* in a filarial nematode residing within one of the *Anopheles coustani* guts (30). Thus, this diagnostic test can detect *Wolbachia* in organisms interacting with *Anophele*s.

Meanwhile, Gomes et al. based their work on a 40-cycle quantitative PCR (qPCR) assay (29). The robustness of this test is not clear, as no raw data were included. Other methods routinely used to detect low-titer *Wolbachia* in insects, like PCR-Southern blotting or amplification of repeated sequences (e.g., the transposases with the highest coverage in the genomic data of Baldini et al. [26]) were never tested on *Wolbachia* sequences found in *Anopheles* (38, 39). Amplification of other *Wolbachia* sequences from putatively infected mosquitos, including *Wolbachia* surface protein and multilocus sequence typing (MLST) genes, has also been challenging (26, 27, 29–31), requiring protocol modifications (30) or the use of more than one mosquito sample (31), and was unsuccessful in some cases (26, 27). Overall, detection of *Wolbachia* sequences in *A. gambiae* by PCR-based methods remains challenging.

**FIG 3:**
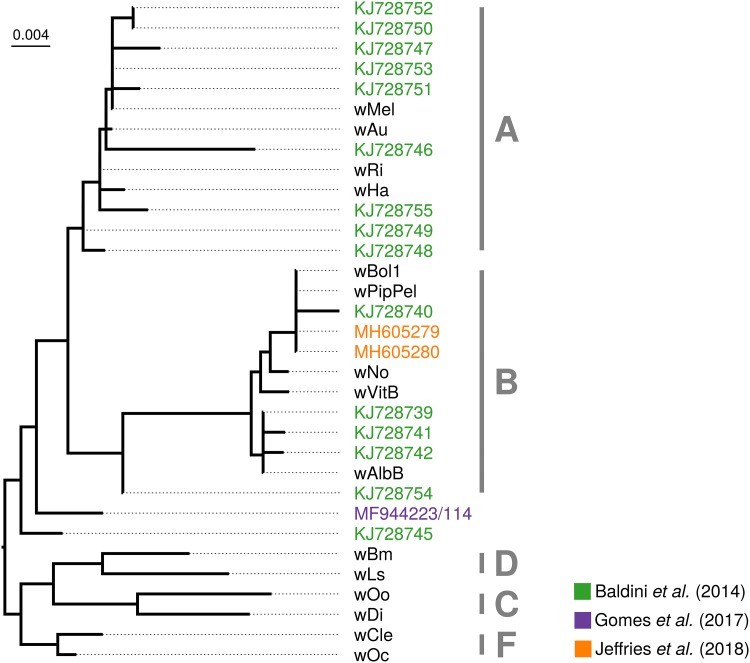
Phylogenetic placement of *Wolbachia* sequences from Anopheles gambiae based on 16S rRNA sequences. Alignment was done with Mafft using the “–auto” option. A maximum-likelihood tree was inferred with automatic model selection in IQ-TREE version 1.62 (60). Origins of sequences are indicated by colors (see the key), and tip names correspond to NCBI accession numbers. All other sequences are reference *Wolbachia* strains. Tentative supergroup affiliations are denoted with capital letters. Please note that the two *Wolbachia* 16S rRNA sequences determined by Gomes et al. (29) overlap. Because the 117-bp overlap regions are 100% identical between these two sequences, we have merged them prior to phylogenetic analysis.

In summary, very few sequence data are available for the putative *Wolbachia* symbiont of *A. gambiae*, despite several attempts at generating and extracting such data. One common feature of all of them is an extremely low titer, at the limit of detection of PCR-based methods. Even from the few data available, it is obvious that there is no single *Wolbachia* strain associated with Anopheles gambiae (Fig. 3). In fact, almost every *Wolbachia* 16S rRNA amplicon and sequence attributed to *A. gambiae* is unique, and their diversity spans at least two *Wolbachia* supergroups (genetic lineages roughly equivalent to those of species in other bacterial genera) (Fig. 3) (40). In combination, we interpret the very low titers and the conflicting phylogenetic affiliations of the sequenced strains as incompatible with the notion of a stable, intraovarially transmitted *Wolbachia* symbiont in *A. gambiae*. However, this conclusion requires alternative explanations for the presence of *Wolbachia* DNA in these malaria mosquitos.

## ORIGIN OF *WOLBACHIA* SEQUENCES IN ANOPHELES GAMBIAE

The presence of *Wolbachia* DNA in A. gambiae samples may be explained not only by a stable *Wolbachia-Anopheles* symbiosis but also in several alternative ways. First, the signal may stem from *Wolbachia* DNA insertions into an insect chromosome (26). Fragments of *Wolbachia* genomes are frequently found within insect genomes (41–43), and the most spectacular case includes a nearly complete genome insertion in Drosophila ananassae (44). This possibility was discussed by Baldini et al., but as the authors point out, the presence of the sequences in only some tissues and their very low titer argue against this hypothesis (26). The second possibility discussed by Baldini and colleagues is the insertion of a *Wolbachia* fragment into the chromosome of another, so-far-unidentified, mosquito-associated microorganism. However, this hypothesis does not help to explain the diversity of *Wolbachia* 16S rRNA sequences found in *Anopheles*.

Another hypothesis explaining the presence of *Wolbachia* sequences in *A. gambiae* tissues is contamination of the mosquito surface or gut. This contamination might come from several sources. First, ectoparasitic mites or midges and endoparasitic nematodes in *Anopheles* may contaminate whole-tissue DNA extracts, as shown by the detection of the *Wolbachia* symbiont of Dirofilaria immitis in an Anopheles coustani DNA preparation (30). However, the presence of unknown symbionts or parasites with novel *Wolbachia* strains is very challenging to test for.

The second possible source of *Wolbachia* contamination is plants. It has been shown that *Wolbachia* can persist in plants on which *Wolbachia*-infected insects feed and then be detected in previously uninfected insects reared on the same plants (reviewed in reference 45). As malaria vectors feed on plant nectar and fruits in the wild, *Wolbachia* DNA traces from these sources may accumulate in their guts. Feeding on *Wolbachia*-infected food may explain encountering *Wolbachia* 16S rRNA in the ovaries, as the adjacent gut can easily be perforated during dissections, releasing content and contaminating other tissues. Again, *Wolbachia* sequences from the gut may also explain the detection of *Wolbachia* sequences in larvae, as eggs and larval habitats may be contaminated with adult feces.

Another possible source of contamination is other insects cohabiting the collection sites. *Culex*, *Aedes*, and other *Anopheles* species can be found in sub-Saharan Africa, and all genera include natural *Wolbachia* hosts. This route of contamination seems especially plausible for mosquito larvae, which are avid predators, attacking other water-inhabiting insects. Moreover, *Wolbachia* 16S rRNA sequences can be detected in water storage containers inhabited by the larvae of various mosquito species (Text S1) and because of this may also be acquired by newly emerging adults and females during egg laying (46). Unfortunately, we have no data on the water composition of the breeding sites of the putative *A. gambiae Wolbachia* carriers, which may explain the *Wolbachia* sequence presence throughout the mosquito life cycle.

Part of the PCR signals observed throughout the studies reporting natural *Wolbachia* infections in *A. gambiae* may be purely technical and arise/spread at the level of DNA extraction and PCR. Although the original contamination likely originates from the field (as each sequenced amplicon has a different sequence), once amplified to high concentration in the lab, the contaminating templates may spread. This is especially true for extractions and PCR amplifications performed under field conditions and for labs that routinely amplify *Wolbachia* from other sources.

The data on natural *Wolbachia* infections in *A. gambiae*, together with similar reports suggesting *Wolbachia* infections in species previously considered uninfected, e.g., *A. stephensi* (47), Anopheles funestus (48), and A. aegypti (see references 47, 49, and 50 but also 51 and 52), should be carefully examined, as all have aquatic, detritus-feeding, and predatory larvae, while adults are terrestrial and can feed on nectar. Thus, bacteria and/or contaminating sequences may spread between these and other organisms sharing the same niches, necessitating studies designed to discern candidates for symbiotic taxa from transient and contaminating bacteria. Sampling of the mosquitos along with their environments and cohabiting species may help to reveal the origin and nature of *Wolbachia* sequences identified in *A. gambiae*.

Importantly, contamination from any of the mentioned sources cannot be ruled out with the data currently available. The previously mentioned sequencing of two *Wolbachia*-positive ovary samples resulted in 571 (out of ∼800,000,000) reads being classified as *Wolbachia* (0.000063%) (26). For a highly sensitive sequencing technique, such as Illumina sequencing, this falls well within the expected coverage of contaminants. Deep shotgun sequencing of eukaryotes usually results in some nontarget sequences from environmental contaminants, and it is unlikely that the *A. gambiae* libraries are an exception (53–55). Contamination stemming from nontarget microbial taxa is especially problematic in low-biomass samples (56), such as single mosquito ovaries. Adding to the difficulty, all of the studies reporting *Wolbachia* from amplicon or metagenomic sequencing do not present negative controls (e.g., sequencing of extraction or blank controls, quantification of microbial taxa, sequencing of mock communities [26, 27, 29–31]). This is not to say that the *Wolbachia* sequences definitely constitute contaminants, but they are simply not discernible from such. In general, the detection of very low-titer *Wolbachia* through highly sensitive methods (nested PCRs, Illumina sequencing) alone is not sufficient to conclude that an intracellular, inherited symbiont is present in a sample.

## EXPECTED FEATURES OF NATURAL *WOLBACHIA* FROM ANOPHELES GAMBIAE

While sequence data alone are insufficient to determine whether *Wolbachia* is a symbiont of Anopheles gambiae and assembly of complete genomes has not been achieved due to low sequence abundance, other hallmarks of symbiotic interactions between the taxa can be used to support this claim.

First, intracellular localization is imperative for *Wolbachia*. The only published image of natural *Wolbachia* infections from *A. gambiae* is a combination of fluorescence *in situ* hybridization (FISH) and immunofluorescence. In this experiment, *Wolbachia* was detected with a combination of a Cy3-labeled probe, anti-Cy3 mouse antibody, and anti-mouse Alexa448 secondary antibody (see Fig. 1 in reference 28). The probe was designed to hybridize within the *w*Spec amplicon region. However, the low resolution of the image and the lack of host membrane staining do not allow us to confirm the *w*Spec intracellular localization (28). The indirect nature of the staining (the RNA probe was detected by primary and secondary antibodies) calls for additional controls acquired with the same microscope settings, and the nature of the findings calls for broader sampling and images at higher magnifications. Electron microscopy showing an immunogold-labeled *Wolbachia* cell or high-resolution FISH combined with membrane staining would provide unequivocal visual evidence for the existence of intracellular *Wolbachia* infections in *A. gambiae*.

Second, *Wolbachia*’s intracellular lifestyle is directly related to its mode of transmission, which is expected to occur from mother to offspring within the mother’s ovaries. In the first study on natural *Wolbachia* in *A. gambiae*, maternal transmission of the detected *w*Spec sequences was also examined. In this experiment, five *w*Spec-positive wild-caught gravid females oviposited in the lab, and their larval progeny was tested for *w*Spec amplification (detected in 56% to 100% of the offspring) (26). However, intraovarial transmission of *Wolbachia* was never explicitly addressed. Surface sterilization of eggs after oviposition would help to determine the transmission mode of these sequences, just as would testing for and excluding horizontal (between larvae or adult to larvae) and paternal *w*Spec sequence transmission. These experiments would help to confirm that *A. gambiae* is infected with an intracellular, transovarially transmitted symbiont and, together with the PCR evidence, diagnose a stable *Wolbachia* infection.

## *WOLBACHIA* SYMBIONTS OF ANOPHELES GAMBIAE AND MALARIA

*Wolbachia* phenotypes similar to those observed in other insect hosts may have a huge impact on wild *Anopheles* populations and malaria transmission. Reproductive manipulations and fitness benefits may increase the proportion of biting females spreading the disease, while pathogen blocking may limit *Plasmodium* prevalence in the wild mosquito populations. Understanding Anopheles gambiae biology is crucial for the design of effective strategies aiming at limiting *Plasmodium* transmission.

Targeted *Wolbachia-*based *Plasmodium* control strategies, similar to the ones used for dengue and Zika virus control, are also exciting prospects. However, they are not reliant on *Wolbachia* symbionts naturally associated with *Anopheles.* Insect populations may equally well be suppressed by the release of males carrying incompatible *Wolbachia* strains by bidirectional CI on an infected population or by unidirectional CI on an uninfected one. The same applies to *Wolbachia*-induced pathogen blocking. Existing initiatives to control dengue and Zika virus with *Wolbachia*-conferred antiviral protection use naturally uninfected Aedes aegypti mosquitos that were artificially transinfected with *Wolbachia* from a different insect species (12). These mosquitos benefit not only from protection by the core and yet-unknown mechanism but also from immune system upregulation caused by a recent transinfection with *Wolbachia* (10). Thus, the *Wolbachia*-based population suppression and disease blocking can work in species not commonly infected with *Wolbachia* in the wild.

The presence of and, subsequently, the *Plasmodium* blocking properties of the presumed natural *Wolbachia* strains in *A. gambiae* remain to be confirmed. Given that *Wolbachia* detection in *A. gambiae* remains challenging (with PCR-based replicate experiments yielding discordant results [29]), it was surprising that two studies have reported negative correlations between the low-titer *Wolbachia* sequences and *Plasmodium* (28, 29). As pathogen protection has been shown to depend on the symbiont titer (57–59) and has so far been detected only in strains exhibiting relatively high bacterial load, it is likely to be absent from *A. gambiae* (31). However, the mechanism of *Plasmodium* blocking by *Wolbachia* may be different than the one characterized for viruses and requires further investigation. Moreover, CI necessary for the spread of *Wolbachia* in artificially infected vector populations was also not detected (28). Reliable protocols for the detection of *Wolbachia* in *A. gambiae*, together with independent repetition efforts, seem necessary to characterize the potential of the putative *A. gambiae* symbionts for their deployment in vector or disease control programs.

In summary, although using *Wolbachia* to fight malaria has been eagerly anticipated, naturally occurring *Wolbachia* strains in *Anopheles* were never an absolute requirement for this to be successful. Even now, their presence, phenotypes, and suitability for deployment in disease control remain to be confirmed. However, they should be studied, as understanding Anopheles gambiae biology and ecology, including its interactions with other micro- and macroscopic organisms, is crucial for designing effective malaria elimination programs.

## CONCLUSIONS

The evidence for natural *Wolbachia* infections in Anopheles gambiae is currently limited to a small number of highly diverse, very low-titer DNA sequences detected in this important malaria vector. Further efforts toward characterization of the interaction between *Wolbachia* sequences and *A. gambiae* are required to establish that this is a true symbiotic association. Demonstrating the presence of intracellular bacterial cells and their intraovarian transmission are prerequisites to diagnose a symbiosis. Additionally, genomic data may shed light on the features of these *Wolbachia* and may reveal the origin of the sequences and the ecological interactions that caused their acquisition by *A. gambiae* mosquitos. Finally, ascertaining phenotypes associated with these *Wolbachia* sequence variants will improve our understanding of Anopheles gambiae biology, and as such inform future strategies aimed at limiting malaria spread and eventual disease eradication. Given that both Shaw et al. and Gomes et al. report the establishment of the *w*Spec-positive *A. gambiae* laboratory colonies (28, 29), the suggested conclusive experiments should be straightforward to perform.

The fact that *Wolbachia* sequences were encountered multiple times by independent groups of researchers clearly indicates present or past, direct or indirect ecological interactions between *Wolbachia* and Anopheles gambiae across Africa. While in-depth investigations of these interactions will be interesting from a basic biology, evolutionary, ecological, and disease control perspective, current data indicate that the postulated natural *Wolbachia* infections in *Anopheles* will be of limited use for application in fighting malaria with *Wolbachia*.
