# Supplementary material for: Is Anopheles gambiae a Natural Host of Wolbachia?
Source: mBio. 2019 Jun 11;10(3):e00784-19. doi: 10.1128/mBio.00784-19 (PMC6561020; doi:10.1128/mBio.00784-19)
Supplement: FIG S1 [file mBio.00784-19-sf001.pdf]

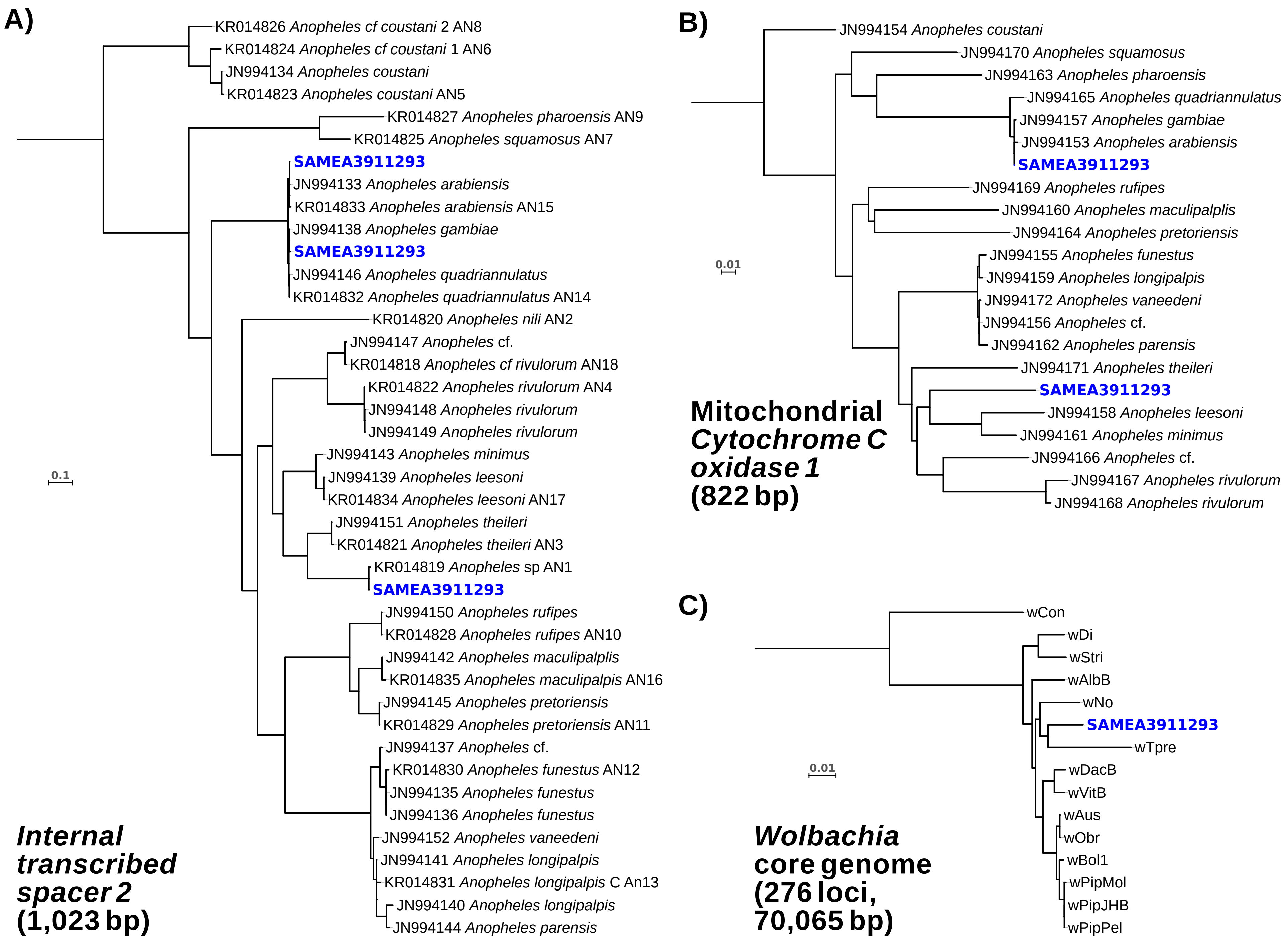
A)
KR014826 Anopheles cf coustani 2 AN8
KR014824 Anopheles cf coustani 1 AN6
JN994134 Anopheles coustani
KR014823 Anopheles coustani AN5
KR014827 Anopheles pharoensis AN9
KR014825 Anopheles squamosus AN7
SAMEA3911293
JN994133 Anopheles arabiensis
KR014833 Anopheles arabiensis AN15
JN994138 Anopheles gambiae
SAMEA3911293
JN994146 Anopheles quadriannulatus
KR014832 Anopheles quadriannulatus AN14
KR014820 Anopheles nili AN2
JN994147 Anopheles cf.
KR014818 Anopheles cf rivulorum AN18
KR014822 Anopheles rivulorum AN4
JN994148 Anopheles rivulorum
JN994149 Anopheles rivulorum
JN994143 Anopheles minimus
JN994139 Anopheles leesoni
KR014834 Anopheles leesoni AN17
JN994151 Anopheles theileri
KR014821 Anopheles theileri AN3
KR014819 Anopheles sp AN1
SAMEA3911293
JN994150 Anopheles rufipes
KR014828 Anopheles rufipes AN10
JN994142 Anopheles maculipalplis
KR014835 Anopheles maculipalpis AN16
JN994145 Anopheles pretoriensis
KR014829 Anopheles pretoriensis AN11
JN994137 Anopheles cf.
KR014830 Anopheles funestus AN12
JN994135 Anopheles funestus
JN994136 Anopheles funestus
JN994152 Anopheles vaneedeni
JN994141 Anopheles longipalpis
KR014831 Anopheles longipalpis C An13
JN994140 Anopheles longipalpis
JN994144 Anopheles parensis
0.1
Internal transcribed spacer 2 (1,023 bp)
B)
JN994154 Anopheles coustani
JN994170 Anopheles squamosus
JN994163 Anopheles pharoensis
JN994165 Anopheles quadriannulatus
JN994157 Anopheles gambiae
JN994153 Anopheles arabiensis
SAMEA3911293
JN994169 Anopheles rufipes
JN994160 Anopheles maculipalplis
JN994164 Anopheles pretoriensis
JN994155 Anopheles funestus
JN994159 Anopheles longipalpis
JN994172 Anopheles vaneedeni
JN994156 Anopheles cf.
JN994162 Anopheles parensis
JN994171 Anopheles theileri
SAMEA3911293
JN994158 Anopheles leesoni
JN994161 Anopheles minimus
JN994166 Anopheles cf.
JN994167 Anopheles rivulorum
JN994168 Anopheles rivulorum
0.01
Mitochondrial Cytochrome C oxidase 1 (822 bp)
C)
wCon
wDi
wStri
wAlbB
wNo
SAMEA3911293
wTpre
wDacB
wVitB
wAus
wObr
wBol1
wPipMol
wPipJHB
wPipPel
0.01
Wolbachia core genome (276 loci, 70,065 bp)
